# Pseudomonas aeruginosa Stimulates Inflammation and Enhances Kaposi’s Sarcoma Herpesvirus-Induced Cell Proliferation and Cellular Transformation through both Lipopolysaccharide and Flagellin

**DOI:** 10.1128/mBio.02843-20

**Published:** 2020-11-10

**Authors:** Ashley Markazi, Paige M. Bracci, Michael McGrath, Shou-Jiang Gao

**Affiliations:** a Cancer Virology Program, UPMC Hillman Cancer Center, Pittsburgh, Pennsylvania, USA; b Department of Microbiology and Molecular Genetics, University of Pittsburgh School of Medicine, Pittsburgh, Pennsylvania, USA; c Department of Epidemiology and Biostatistics, University of California at San Francisco, San Francisco, California, USA; d The AIDS and Cancer Specimen Resource and Department of Laboratory Medicine, Pathology, and Medicine, University of California at San Francisco, San Francisco, California, USA; University of North Carolina, Chapel Hill

**Keywords:** Kaposi’s sarcoma-associated herpesvirus, KSHV, Kaposi’s sarcoma, *Pseudomonas aeruginosa*, lipopolysaccharide, LPS, flagellin, inflammation, MAPK, cellular transformation

## Abstract

Inflammation triggered by innate immunity promotes carcinogenesis in cancer. Kaposi’s sarcoma (KS), a hyperproliferative and inflammatory tumor caused by Kaposi’s sarcoma-associated herpesvirus (KSHV) infection, is the most common cancer in AIDS patients. KSHV infection sensitizes cells to pathogen-associated molecular patterns (PAMPs). We examined the role of Pseudomonas aeruginosa, an opportunistic bacterium that can affect AIDS patients, in inflammation and cell proliferation of KSHV-transformed cells. P. aeruginosa stimulation increased cell proliferation and efficiency of colony formation in soft agar of KSHV-transformed rat primary mesenchymal precursor (KMM) cells but had no significant effect on the untransformed (MM) cells. P. aeruginosa stimulation also increased cell proliferation of KSHV-infected human B cells, BJAB, but not the uninfected cells. Mechanistically, P. aeruginosa stimulation resulted in increased inflammatory cytokines and activation of p38, ERK1/2, and JNK mitogen-activated protein kinase (MAPK) pathways in KMM cells while having no obvious effect on MM cells. P. aeruginosa induction of inflammation and MAPKs was observed with and without inhibition of the Toll-like receptor 4 (TLR4) pathway, while a flagellin-deleted mutant of P. aeruginosa required a functional TLR4 pathway to induce inflammation and MAPKs. Furthermore, treatment with either lipopolysaccharide (LPS) or flagellin alone was sufficient to induce inflammatory cytokines, activate MAPKs, and increase cell proliferation and efficiency of colony formation in soft agar of KMM cells. These results demonstrate that both LPS and flagellin are PAMPs that contribute to P. aeruginosa induction of inflammation in KSHV-transformed cells. Because AIDS-KS patients are susceptible to P. aeruginosa infection, our work highlights the preventive and therapeutic potential of targeting P. aeruginosa infection in these patients.

## Abstract (precis)

Kaposi’s sarcoma (KS), caused by infection with Kaposi’s sarcoma-associated herpesvirus (KSHV), is one of the most common cancers in AIDS patients. KS is a highly inflammatory tumor, but how KSHV infection induces inflammation remains unclear. We have previously shown that KSHV infection upregulates Toll-like receptor 4 (TLR4), sensitizing cells to lipopolysaccharide (LPS) and Escherichia coli. In the current study, we examined the role of Pseudomonas aeruginosa, an opportunistic bacterium that can affect AIDS patients, in inflammation and cell proliferation of KSHV-transformed cells. P. aeruginosa stimulation increased cell proliferation, inflammatory cytokines, and activation of growth and survival pathways in KSHV-transformed cells through two pathogen-associated molecular patterns, LPS and flagellin. Because AIDS-KS patients are susceptible to P. aeruginosa infection, our work highlights the preventive and therapeutic potential of targeting P. aeruginosa infection in these patients.

## INTRODUCTION

Inflammation is the immune system’s response to tissue damage or microbial infection. When unregulated, inflammation can cause or exacerbate carcinogenesis (1). For example, the continuous induction of inflammatory cytokines and cytotoxic mediators, such as reactive oxygen or nitrogen species, can, over time, cause damage to the cellular genome, resulting in cellular mutations that lead to dysregulated cell proliferation and cancer development (2). Inflammatory cytokines can further enhance cancer progression by activating pro-oncogenic and -survival cellular pathways, including NF-κB and STAT3 pathways (3). By further elucidating the mechanisms by which inflammation promotes cancer cell growth, proliferation, and survival, we can gain important insights into the mechanism of oncogenesis and identify novel therapeutic targets and biomarkers.

Interestingly, patients with HIV/AIDS are at a much higher risk for developing AIDS-associated cancer (AAC) than those with healthy immune systems (4). HIV patients are 500 times more likely to develop Kaposi’s sarcoma (KS), 12 times more likely to be diagnosed with non-Hodgkin’s lymphoma, and, among women, 3 times more likely to be diagnosed with cervical cancer (4). Moreover, HIV patients are at a higher risk for non-AIDS-defining cancer (NADC) as well, including anal cancer, liver cancer, lung cancer, and Hodgkin’s lymphoma (5). HIV enhances cellular transformation through its protein, Tat, which represses tumor suppressor gene p53, promotes cell cycle progression, and induces inflammation (6, 7). For cancers caused by infections of oncogenic viruses, HIV regulates both the replication of these viruses and the progression of their associated cancers (8). For example, HIV-encoded products Tat, Nef, and Vpr regulate KSHV replication and the functions of KSHV genes, resulting in enhanced cell migration, invasion, and angiogenesis (9–15). Moreover, long-term use of antiretroviral drugs in HIV patients is associated with an elevated risk of several cancers (16).

A number of other mechanisms also contribute to the chronic inflammation and increased cancer risk in HIV-infected patients. One of these factors is immunodeficiency associated with HIV infection, such as declined CD4^+^ T cell count (17). Because CD4^+^ T cells stimulate B cells and CD8^+^ T cells, patients with a low CD4^+^ T cell count are less capable of eliminating infections, including viruses, bacteria, and fungi, thereby increasing pathogen-associated molecular patterns (PAMPs) (18), which continuously stimulate immune and cancer cells to secrete inflammatory cytokines, resulting in chronic inflammation. Indeed, many reports show that a lower CD4^+^ cell count is correlated with an increase in inflammatory cytokines (19). Moreover, chronic stress, such as metabolic stress of the immune and cancer cells induced by HIV infection, as well as the long-term use of antiretroviral drugs, triggers damage-associated molecular patterns (DAMPs), which further promote chronic inflammation in the same fashion as PAMPs (20).

KS, the most common cancer in HIV-infected patients, is a hyperproliferative and inflammatory cancer caused by infection with Kaposi’s sarcoma-associated herpesvirus (KSHV) (21). KSHV infection provides an excellent model for examining the complex interactions of HIV, a cancer-causing virus (KSHV), innate immunity, inflammation, and cancer. KSHV encodes numerous genes that directly contribute to cellular transformation (21), and KSHV infection alone is sufficient to induce inflammatory cytokines, which can stimulate cell proliferation and survival and regulate KSHV replication (22–28). Furthermore, we have shown that KSHV infection sensitizes the infected cells to PAMPs, leading to the activation of Toll-like receptor 4 (TLR4) and alternative complement pathways, which induce inflammatory cytokines and promote cell survival, proliferation, and cellular transformation (22, 29). The Escherichia coli- and lipopolysaccharide (LPS)-activated TLR4 pathway stimulates cell proliferation, cellular transformation, and tumorigenesis by increasing interleukin-6 (IL-6) expression to activate the STAT3 pathway (22). Indeed, in a recent study, we showed the impoverishment of oral microbial diversity and enrichment of specific microbiota in oral KS in HIV-infected patients (30). However, the precise mechanism of how specific microbiota promote KS remains to be elucidated.

Pseudomonas aeruginosa is normally considered a commensal bacterium. However, P. aeruginosa can cause severe infection in individuals with immunosuppression (31). HIV/AIDS patients with CD4^+^ T cell counts below 200 cell/mm^3^ are at a significantly higher risk for P. aeruginosa infection (32). P. aeruginosa consists of PAMPs, such as LPS and flagellin, which activate TLR4 and TLR5, respectively (33). Hence, P. aeruginosa infection might induce inflammatory cytokines of KSHV-infected cells and promote cell proliferation and cellular transformation.

In the current study, we analyzed the effects of P. aeruginosa on cell proliferation and cellular transformation in a KS-like model of KSHV-induced cellular transformation of rat primary embryonic metanephric mesenchymal precursor cells (MM) (34). We observed that P. aeruginosa stimulation increased both cell proliferation and cellular transformation in KSHV-transformed MM cells (KMM) yet had no significant effect on MM cells. Moreover, we observed similar results of increased cell proliferation in a KSHV-infected human B cell line, KSHV-BJAB, compared to the BJAB uninfected control. In KMM cells, P. aeruginosa stimulation resulted in increased expression of inflammatory cytokines and activation of p38, ERK1/2, and JNK pathways. Interestingly, we observed the induction of inflammatory cytokines and activation of the p38 and ERK1/2 pathways, even after the inhibition of the TLR4 pathway in KMM cells stimulated by P. aeruginosa, and that this effect disappeared when KMM cells were stimulated with a flagellin-deleted mutant of P. aeruginosa, indicating that P. aeruginosa mediated inflammation and cellular transformation of KSHV-transformed cells through both LPS and flagellin.

## RESULTS

### P. aeruginosa stimulation enhances cell proliferation and cellular transformation of KMM cells but has no significant effect on MM cells.

**FIG 1:**
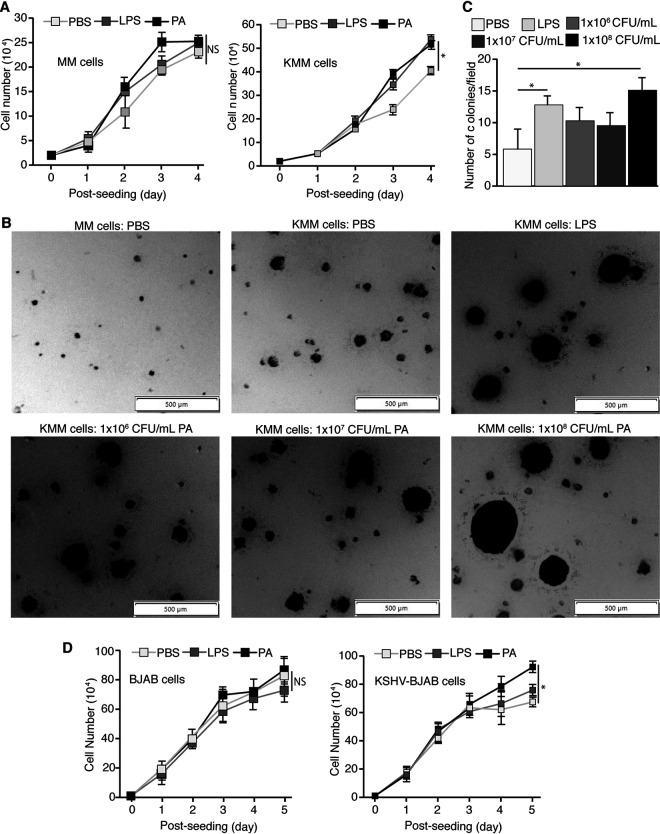
P. aeruginosa (PA) stimulation enhances cell proliferation and cellular transformation of KSHV-infected cells but has no significant effect on the uninfected cells. (A) Cell proliferation of MM and KMM cells treated with PBS, 1 μg/ml LPS, or 1 × 10^7^ CFU/ml P. aeruginosa (ATCC 15442), analyzed by cell counting. (B and C) Formation of colonies of KMM cells in soft agar treated with PBS, 1 μg/ml LPS, or 1 × 10^6^ to 1 × 10^8^ CFU/ml P. aeruginosa (ATCC 15442), shown by representative pictures (B) and results of statistical analysis from 3 wells, each with 5 representative fields (C). (D) Cell proliferation of BJAB and KSHV-BJAB cells treated with PBS, 1 μg/ml LPS, or 1 × 10^7^ CFU/ml P. aeruginosa (ATCC 15442), analyzed by cell counting. *, *P* ≤ 0.05. NS, not significant. PA, P. aeruginosa.

To examine the effect of P. aeruginosa on the proliferation of KSHV-transformed cells, we treated the cells with 1 × 10^7^ CFU/ml P. aeruginosa (ATCC 15442) or 1 μg/ml LPS. P. aeruginosa increased the proliferation of KMM cells but did not have any significant effect on MM cells (Fig. 1A). Similar results were observed with LPS, as expected (22). Both P. aeruginosa and LPS also increased the sizes and efficiency of colony formation in soft agar of KMM cells (Fig. 1B and C). As previously reported, MM cells did not form any significant colonies (34). These results indicated that, similar to LPS, P. aeruginosa stimulated the proliferation and cellular transformation of KMM cells (22). To assess the effects of P. aeruginosa and LPS stimulation on KSHV-infected human B cells, we treated BJAB and KSHV-BJAB cells with 1 × 10^7^ CFU/ml P. aeruginosa (ATCC 15442) or 1 μg/ml LPS. Although less pronounced than that in KMM cells, P. aeruginosa stimulation also increased proliferation of KSHV-BJAB cells while having no significant effects in BJAB cells (Fig. 1D). Because KMM cells can form colonies in soft agar, permitting the evaluation of the transforming potential of the cells, we chose to further examine the effect of P. aeruginosa on KMM cells and the control MM cells in subsequent experiments (34).

### P. aeruginosa stimulation increases the expression levels of inflammatory cytokines in KMM cells while having minimal effect on MM cells.

**FIG 2:**
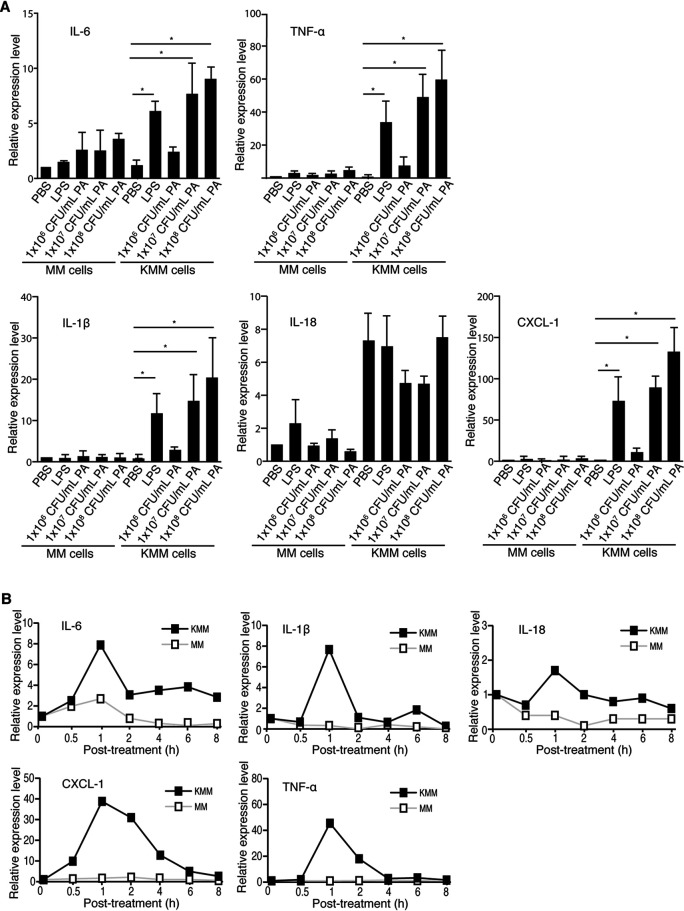
P. aeruginosa (PA) stimulation increases the expression of inflammatory cytokines in KMM cells while having minimal effect on MM cells. (A) MM and KMM cells were treated with PBS, 1 μg/ml LPS, or 1 × 10^6^ to 1 × 10^8^ CFU/ml P. aeruginosa (ATCC 15442) for 1 h and analyzed for cytokines IL-6, TNF-α, IL-1β, IL-18, and CXCL-1 by qRT-PCR. (B) MM and KMM cells were treated with PBS or 1 × 10^7^ CFU/ml P. aeruginosa (ATCC 15442) at the specified time points and analyzed for cytokines IL-6, TNF-α, IL-1β, IL-18, and CXCL-1 by qRT-PCR. *, *P* ≤ 0.05.

We previously showed that purified E. coli LPS induced the inflammatory cytokines interleukin-6 (IL-6), IL-1β, and IL-18 in KMM cells but had only a weak effect on MM cells. P. aeruginosa (ATCC 15442) stimulation resulted in higher mRNA levels of IL-6 and IL-1β but had no significant effect on IL-18 in KMM cells (Fig. 2A). Additionally, we analyzed the cytokines tumor necrosis factor alpha (TNF-α) and CXCL-1, as P. aeruginosa increased levels of these inflammatory cytokines in mice (35, 36). P. aeruginosa stimulation also resulted in higher mRNA levels of TNF-α and CXCL-1 in KMM cells (Fig. 2A). In contrast, cytokines IL-6, IL-1β, IL-18, TNF-α, and CXCL-1 were not significantly upregulated in MM cells by P. aeruginosa (Fig. 2A).

We further examined the induction kinetics of inflammatory cytokines in KMM cells by P. aeruginosa by stimulating the cells with 1 × 10^7^ CFU/ml P. aeruginosa (ATCC 15442) and analyzing them at 0, 0.5, 1, 2, 4, 6, and 8 h poststimulation. IL-6, IL-1β, TNF-α, and CXCL-1 had the highest mRNA levels at 1 h after P. aeruginosa stimulation in KMM cells (Fig. 2B). No significant induction of inflammatory cytokines at different time points in MM cells was observed following P. aeruginosa stimulation (Fig. 2B).

### P. aeruginosa stimulation activates the MAPK pathways in KMM cells but has no obvious effect in MM cells.

**FIG 3:**
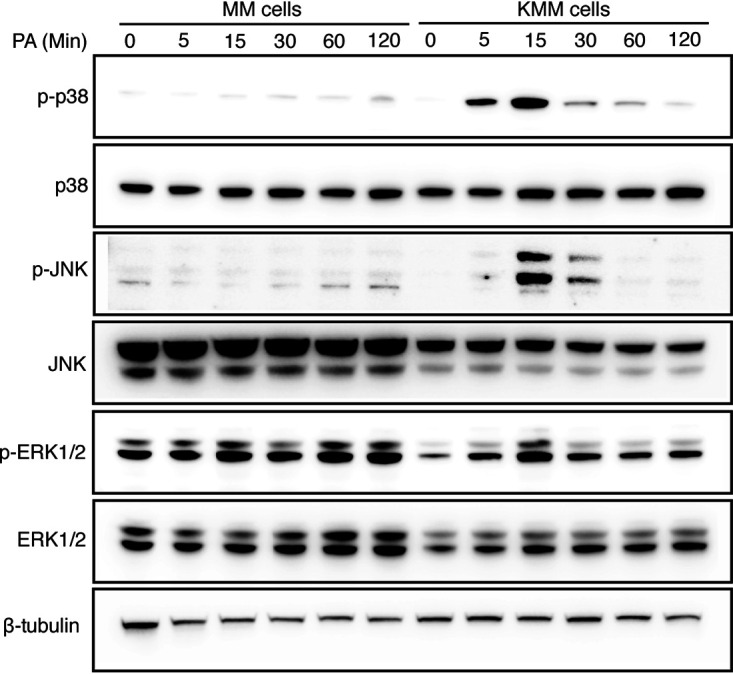
P. aeruginosa (PA) stimulation activates the p38, JNK, and ERK1/2 MAPK pathways in KMM cells but has no obvious effect on MM cells. MM and KMM cells were stimulated with 1 × 10^7^ CFU/ml P. aeruginosa (ATCC 15442) for the specific times and analyzed by Western blotting.

The p38, ERK1/2, and JNK mitogen-activated protein kinase (MAPK) pathways have been implicated in the induction of inflammatory cytokines and are commonly activated in cancer cells (37). We examined the activation of these pathways following stimulation with 1 × 10^7^ CFU/ml P. aeruginosa (ATCC 15442) in MM and KMM cells. We detected the activation of p38, ERK1/2, and JNK pathways, which peaked at 15 min after P. aeruginosa stimulation in KMM cells (Fig. 3). In contrast, no increased activation of p38, ERK1/2, or JNK was observed in MM cells (Fig. 3).

### P. aeruginosa stimulation increases the expression of inflammatory cytokines by both TLR4-dependent and -independent mechanisms in KSHV-transformed cells.

**FIG 4:**
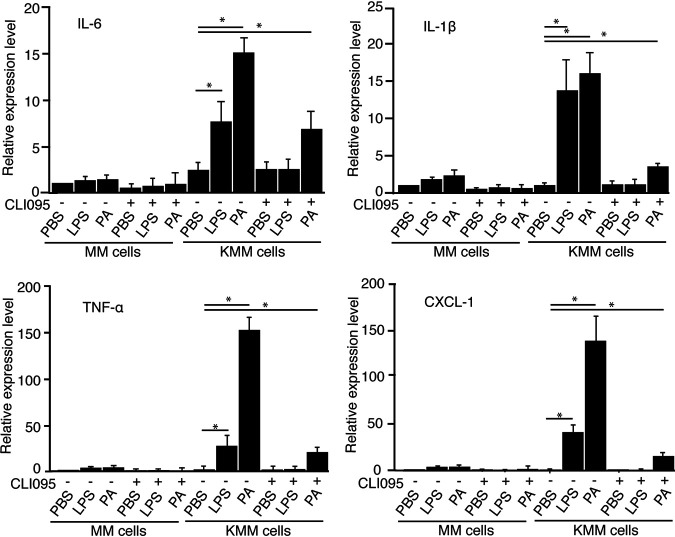
P. aeruginosa (PA) stimulation increases the expression of inflammatory cytokines by both TLR4-dependent and -independent mechanisms in KSHV-transformed cells. MM and KMM cells were treated with 10 μg/ml TLR4 inhibitor (CLI095) for 1 h and then treated with PBS, 1 μg/ml LPS, or 1 × 10^7^ CFU/ml P. aeruginosa (ATCC 15442) for 1 h and analyzed for the expression of cytokines by RT-qPCR. *, *P* ≤ 0.05.

We have previously shown that LPS can induce inflammatory cytokines in KMM cells. Since P. aeruginosa contains other PAMPs in addition to LPS, to determine if other P. aeruginosa PAMPs also contributed to the P. aeruginosa-induced inflammation in KMM cells, we stimulated KMM cells with 1 × 10^7^ CFU/ml P. aeruginosa (ATCC 15442) in the presence of 10 μg/ml TLR4 inhibitor CLI095. The levels of induced proinflammatory cytokines IL-6, IL-1β, TNF-α, and CXCL-1 by P. aeruginosa were significantly decreased in KMM cells by CLI095; however, they remained significantly higher than those of the unstimulated KMM cells (Fig. 4). As expected, induction of inflammatory cytokines by LPS was completely blocked by CLI095 in KMM cells, with levels similar to those of the unstimulated cells or cells treated with CLI095 alone (Fig. 4). No obvious change of inflammatory cytokines in MM cells was observed under these treatments. These results indicated that, besides LPS, another P. aeruginosa PAMP(s) also contributes to P. aeruginosa-induced inflammation in KMM cells.

### P. aeruginosa flagellin contributes to P. aeruginosa induction of inflammatory cytokines in KMM cells but has no significant effect on MM cells.

**FIG 5:**
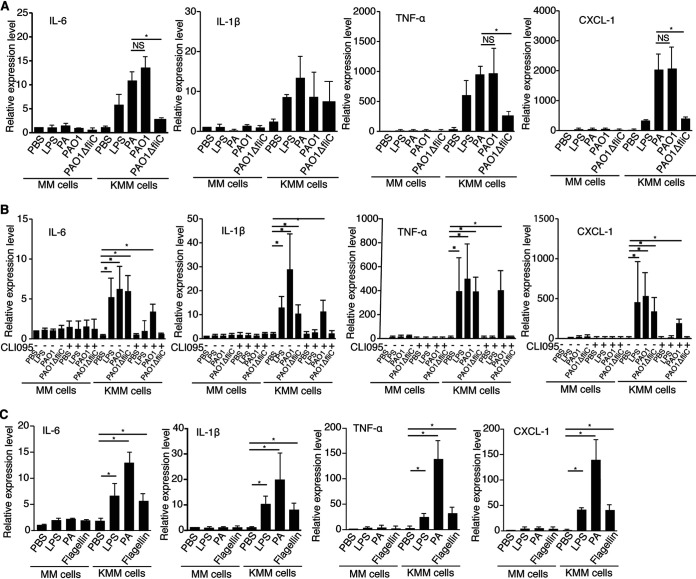
P. aeruginosa (PA) flagellin contributes to P. aeruginosa induction of inflammatory cytokines in KMM cells but has no significant effect on MM cells. (A) MM and KMM cells were treated with PBS, 1 μg/ml LPS, 1 × 10^7^ CFU/ml P. aeruginosa (ATCC 15442), 1 × 10^7^ CFU/ml PAO1, or 1 × 10^7^ CFU PAO1Δ*fliC* strain for 1 h and analyzed for the expression of cytokines by qRT-PCR. (B) MM and KMM cells were treated with 10 μg/ml TLR4 inhibitor (CLI095) for 1 h and then treated with PBS, 1 × 10^7^ CFU/ml wild-type PAO1, or 1 × 10^7^ CFU/ml PAO1Δ*fliC* strain for 1 h and analyzed for the expression of cytokines by qRT-PCR. (C) MM and KMM cells were treated with PBS, 1 μg/ml LPS, 1 × 10^7^ CFU/ml P. aeruginosa (ATCC 15442), or 0.3 μg/ml P. aeruginosa flagellin for 1 h and analyzed for the expression of cytokines by qRT-PCR. *, *P* ≤ 0.05. NS, not significant.

As flagellin is P. aeruginosa’s second most immunogenic PAMP, we stimulated KMM cells with a P. aeruginosa strain with the *fliC* gene deleted from its genome, the PAO1Δ*fliC* strain, which rendered it defective in flagellin expression, and its parallel wild type, P. aeruginosa PAO1 (38). While PAO1 and PAO1Δ*fliC* strains at 1 × 10^7^ CFU/ml induced inflammatory cytokines in KMM cells, the levels of induction were significantly lower in KMM cells stimulated with the PAO1Δ*fliC* than PAO1 strain (Fig. 5A). As expected, 1 × 10^7^ CFU/ml PAO1 induced inflammatory cytokines at levels similar to 1 × 10^7^ CFU/ml P. aeruginosa (ATCC 15442) (Fig. 5A). We then treated MM and KMM cells with CLI095 for 1 h before stimulating with the PAO1 or PAO1Δ*fliC* strain or LPS. Similar to P. aeruginosa (ATCC 15442), the PAO1 induction of inflammatory cytokines was reduced in KMM cells by a TLR4 inhibitor, CLI095, but remained at significantly higher levels than those in the unstimulated cells (Fig. 5B). In contrast, PAO1Δ*fliC* strain induction of inflammatory cytokines was completely abolished by CLI095 (Fig. 5B). No significant effect was observed on MM cells with either the PAO1Δ*fliC* or PAO1 strain (Fig. 5B).

To further confirm the role of flagellin in P. aeruginosa induction of inflammation in KMM cells, we stimulated the cells with 0.3 μg/ml purified P. aeruginosa flagellin. Treatment with purified P. aeruginosa flagellin alone was sufficient to induce inflammatory cytokines in KMM cells at levels similar to those of LPS alone (Fig. 5C). In contrast, purified P. aeruginosa flagellin stimulation had no significant effect on MM cells (Fig. 5C).

Taken together, these results indicated that flagellin contributed to P. aeruginosa induction of inflammatory cytokines in KMM cells. Hence, at least LPS and flagellin contributed to P. aeruginosa induction of inflammation in KMM cells.

### P. aeruginosa flagellin activates p38 and ERK1/2 pathways in KMM cells but has no obvious effect on MM cells.

**FIG 6:**
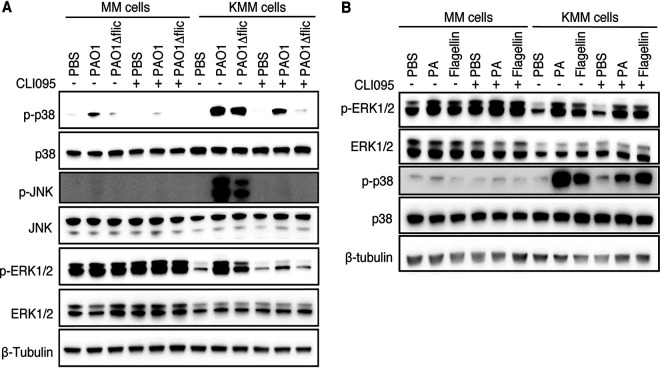
P. aeruginosa-flagellin induces p38 and ERK1/2 activation in KMM cells but has no obvious effect on MM cells. (A) MM and KMM cells were treated for 1 h with 10 μg/ml TLR4 inhibitor CLI095 and then treated with PBS, 1 × 10^7^ CFU/ml wild-type PAO1, or 1 × 10^7^ CFU/ml PAO1Δ*fliC* strain for 15 min and analyzed for the activation of p38, ERK1/2, and JNK pathways by Western blotting. (B) MM and KMM cells were treated for 1 h with 10 μg/ml TLR4 inhibitor CLI095 and then treated for 15 min with PBS, 1 × 10^7^ CFU/ml P. aeruginosa (PA), or 0.3 μg/ml P. aeruginosa flagellin and then analyzed for the activation of p38 and ERK1/2 pathways by Western blotting.

The results described above indicated that the patterns of inflammatory cytokines induced by LPS and flagellin were not entirely the same, which could be due to distinct activation of the signaling pathways by the two PAMPs. While all three MAPK pathways were activated in KMM cells by PAO1, the activation levels were reduced in cells stimulated with the PAO1Δ*fliC* strain (Fig. 6A). Treatment with TLR4 inhibitor CLI095 reduced PAO1 activation of p38 and ERK1/2 pathways but completely abolished that of JNK pathway and PAO1Δ*flliC* strain activation of all three MAPK pathways (Fig. 6A). These results indicated that LPS mediated P. aeruginosa activation of all three pathways while flagellin mediated the activation of p38 and ERK1/2 pathways. We then investigated whether flagellin alone was sufficient to activate the p38 and ERK1/2 pathways in KMM cells by treating the cells with purified P. aeruginosa flagellin. As expected, flagellin alone indeed activated the p38 and ERK1/2 pathways, and treatment with CLI095 had no effect on flagellin activation of these two pathways, although the reduction of ERK1/2 activation by CLI095 in P. aeruginosa-stimulated KMM cells was less pronounced in this experiment, indicating possible variation of P. aeruginosa LPS effect on ERK1/2 activation (Fig. 6B). Together, these results indicated that flagellin contributed to P. aeruginosa activation of p38 and ERK1/2 pathways, and LPS contributed to P. aeruginosa activation of all three MAPK pathways in KMM cells.

### P. aeruginosa flagellin stimulation increases cell proliferation and cellular transformation of KMM cells but has no significant effect on MM cells.

**FIG 7:**
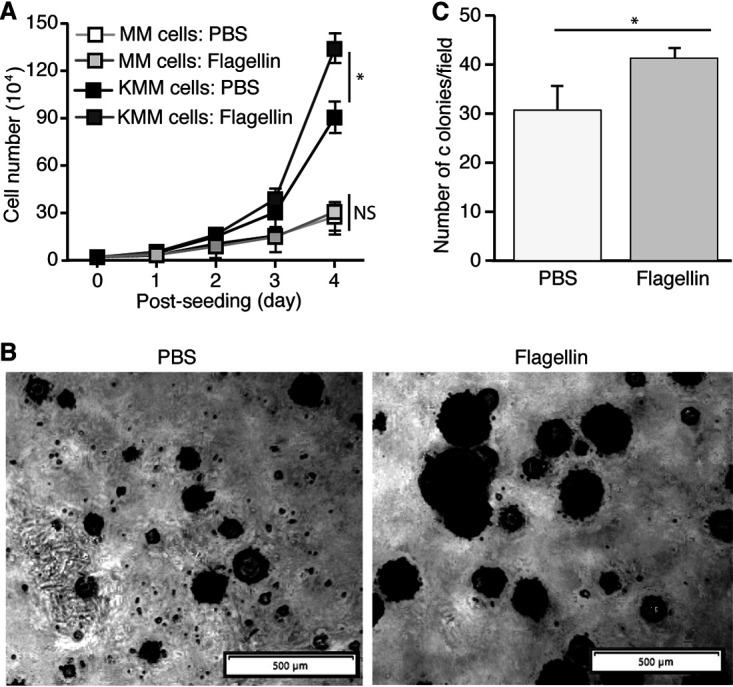
P. aeruginosa flagellin stimulation increases cell proliferation and cellular transformation of KMM cells but has no significant effect on MM cells. (A) Cell proliferation of MM and KMM cells treated with PBS or 1 μg/ml P. aeruginosa flagellin. (B and C) Formation of colonies of KMM cells in soft agar treated with PBS or 1 μg/ml P. aeruginosa flagellin, shown by representative pictures (B) and results of statistical analysis from 3 wells, each with 5 representative fields (C). *, *P* ≤ 0.05. NS, not significant.

Because flagellin participated in P. aeruginosa-induced inflammation and activation of MAPK pathways, we examined whether flagellin could promote KSHV-induced cell proliferation and cellular transformation. KMM cells were treated with purified P. aeruginosa flagellin or phosphate-buffered saline (PBS) as a control and analyzed for cell proliferation. Treatment with P. aeruginosa flagellin alone was sufficient to induce faster cell proliferation in KMM cells than in the untreated cells (Fig. 7A). In contrast, P. aeruginosa flagellin had no effect on the proliferation of MM cells (Fig. 7A).

We further examined the effect of flagellin on cellular transformation of KMM cells. Treatment with purified P. aeruginosa flagellin increased the numbers and sizes of colonies of KMM cells compared to those of the untreated cells (Fig. 7B and C). MM cells did not form any observable colonies in soft agar in both flagellin-treated and untreated cells (results not shown).

### Simultaneous inhibition of p38 and ERK1/2 pathways decreases flagellin-induced inflammation and cell proliferation of KMM cells but has no obvious effect on MM cells.

**FIG 8:**
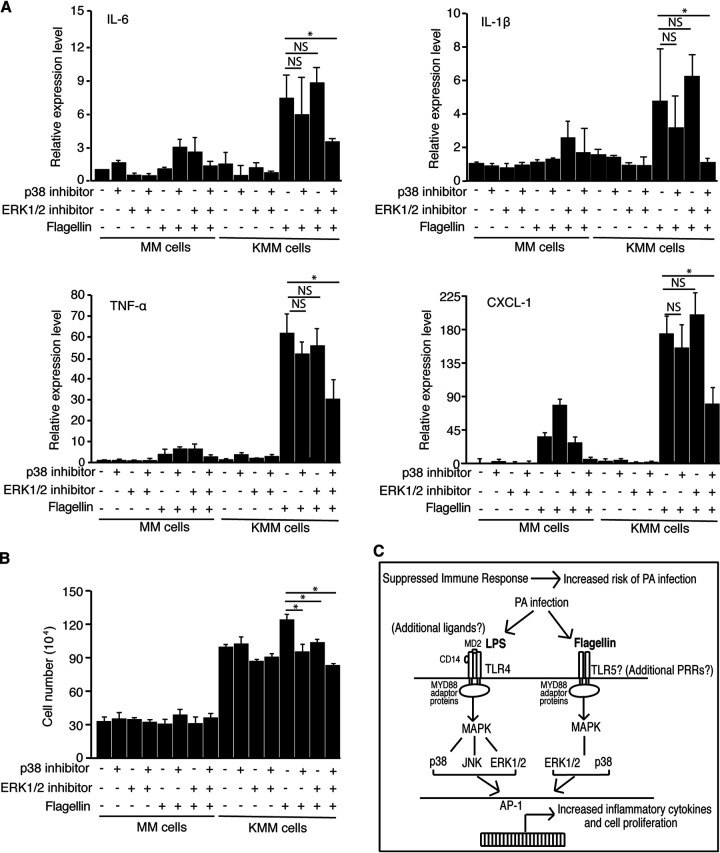
Simultaneous inhibition of p38 and ERK1/2 pathways decreases flagellin-induced inflammation and cell proliferation of KMM cells but has no obvious effect on MM cells. (A) MM and KMM cells were treated for 1 h with PBS, 10 μM p38 inhibitor (SB 203580), 10 μM ERK1/2 inhibitor (U0126), or a combination of 10 μM p38 inhibitor and 10 μM ERK1/2 inhibitor. Cells were then treated with PBS or 0.3 μg/ml flagellin for 1 h and analyzed for cytokines by qRT-PCR. (B) MM and KMM cells were treated for 1 h with PBS, 10 μM p38 inhibitor (SB 203580), 10 μM ERK1/2 inhibitor (U0126), or a combination of 10 μM p38 inhibitor and 10 μM ERK1/2 inhibitor. Cells were then treated with 1 μg/ml flagellin and examined for cell number at day 3 posttreatment. (C) Schematic illustration summarizing P. aeruginosa ligands, and their receptors and activated downstream pathways, that might enhance inflammation and cell proliferation of KSHV-transformed cells during P. aeruginosa infection. *, *P* ≤ 0.05. NS, not significant.

To determine whether p38 and ERK1/2 pathways mediated flagellin-induced inflammation in KMM cells, we stimulated the cells with P. aeruginosa flagellin in the presence of specific inhibitors of these pathways. p38 inhibitor (SB203580) or ERK1/2 inhibitor (U0126) alone had no effect on flagellin induction of inflammatory cytokines (Fig. 8A). However, treatment with both inhibitors significantly decreased the flagellin induction of all inflammatory cytokines (Fig. 8A), confirming the important roles of p38 and ERK1/2 pathways in flagellin induction of inflammatory cytokines. Hence, both p38 and ERK1/2 pathways mediated flagellin induction of inflammatory cytokines.

We further assessed the roles of p38 and ERK1/2 MAPK pathways on flagellin-induced cell proliferation. While P. aeruginosa flagellin stimulated the proliferation of KMM cells by 20%, SB203580 or U0126 alone or in combination completely abolished this effect (Fig. 8B). SB203580 or U0126 alone or in combination had no significant effect on the proliferation of MM cells (Fig. 8B). These results indicated that both p38 and ERK1/2 MAPK pathways were essential for flagellin-stimulated proliferation of KMM cells.

## DISCUSSION

It has been reported that the diversity of overall bacterial species is decreased while the number of pathogenic species is increased in the microbiomes of immunosuppressed patients compared to that in healthy individuals (39). The effects of pathogenic bacteria on cancer pathology have been studied extensively in numerous cancers (40). Pathogenic bacteria such as Fusobacterium nucleatum, Helicobacter pylori, and *Salmonella* spp. can exacerbate cancer pathogenesis by producing bacterial toxin and effector proteins that induce host cell damage and interfere with cell signaling pathways involved in cell proliferation (40). Additionally, bacterial metabolic products, including short-chain fatty acids, can reactivate oncogenic viruses, such as Epstein-Barr virus and KSHV, resulting in dissemination of cancer cells (41). Our study specifically focuses on bacterial PAMPs, which activate TLRs, resulting in prosurvival pathway activation and increased cell proliferation (22).

In the current study, we investigated the role of P. aeruginosa in KSHV induction of chronic inflammation and KSHV-induced cell proliferation and cellular transformation. P. aeruginosa infection can occur in HIV-infected patients and can cause serious complications in immunocompetent hosts despite being a commensal bacterium (32). The results showed that P. aeruginosa stimulation enhanced cell proliferation, and the sizes and efficiency of colony formation in soft agar of KSHV-transformed cells, but had no effect on the uninfected primary cells. Mechanistically, P. aeruginosa stimulation increased the expression of inflammatory cytokines and activated multiple MAPK pathways. Importantly, besides LPS, we found that P. aeruginosa flagellin also contributed to the induction of inflammatory cytokines and cell proliferation of KSHV-transformed cells, and that LPS and flagellin differentially activated MAPK pathways, but both induced similar profiles of inflammatory cytokines (Fig. 8C). Specifically, LPS activated p38, JNK, and ERK1/2 pathways while flagellin activated the p38 and ERK1/2 pathways, despite both PAMPs inducing similar levels of inflammatory cytokines IL-6, IL-1β, TNF-α, and CXCL-1. Our results indicate that KSHV-transformed cells are more susceptible to P. aeruginosa-induced inflammation through the PAMPs LPS and flagellin than the uninfected primary cells. It would be interesting to further confirm the roles of P. aeruginosa in KS development in AIDS-KS patients.

It has been shown that TLR activation leads to activation of downstream prosurvival pathways, resulting in increased cancer pathogenesis (42, 43). We previously showed that KSHV infection upregulated numerous TLRs, including TLR4 and TLR5 (22). LPS and flagellin are highly immunogenic ligands for TLR4 and TLR5, respectively (33). Indeed, TLR4 was upregulated close to 50-fold in KSHV-transformed cells, sensitizing the cells to TLR4 ligands (22). Activation of the TLR4 pathway by E. coli and LPS stimulated cell proliferation, cellular transformation, and tumorigenesis by increasing IL-6 expression to activate the STAT3 pathway (22). In this study, we demonstrate that both P. aeruginosa LPS and flagellin activate the MAPK pathways and that inhibition of MAPK pathways decreases expression of inflammatory cytokines, cell proliferation, and cellular transformation (Fig. 8C).

We previously demonstrated that LPS stimulation increased cytokines IL-1β, IL-6, and IL-18 in KMM cells (22). In our current study, we showed that P. aeruginosa stimulation increased cytokines, including IL-1β, IL-6, TNF-α, and CXCL-1, in KMM cells. Inflammatory cytokines such as TNF-α act synergistically with IL-6 to activate the STAT3 pathway (22, 44). Surprisingly, we did not see an increase in IL-18, despite previous studies reporting IL-18 upregulation correlating with P. aeruginosa infection (45, 46). It is possible that we had missed the peak time point for the induction of IL-18, as we focused on the effect of acute stimulation. As previously reported, we confirmed the induction of TNF-α and CXCL-1 by P. aeruginosa (35, 36, 47). Our results showed that both P. aeruginosa LPS and flagellin contributed to the induction of IL-1β, IL-6, TNF-α, and CXCL-1. It would be interesting to further assess the specific effects of LPS and flagellin in stimulating specific cytokines and their downstream inflammatory pathways in future studies.

The relationship between TLR activation and cancer cell proliferation has been extensively studied (42). Studies have demonstrated that LPS- and flagellin-induced inflammation increases cell proliferation in several cancer cell lines (13, 48, 49). Moreover, some studies suggest that LPS and flagellin blood levels are correlated with a higher cancer risk (50, 51). However, contradictory studies show decreased cell proliferation in LPS- or flagellin-stimulated cells (52–54). In fact, bacteria and the PAMPS have been studied as antitumor agents (55, 56). It is worth mentioning that multiple factors might affect how cells respond to TLR activation. The differences in cell response to TLR activation may depend on the cellular location of the TLRs, as increased TLR expression in the cytoplasm may result in more chronic inflammation and cell proliferation while TLR expression on the cell membrane may result in antitumor effects (57). The proportions of the types of TLRs expressed (TLR2 versus TLR4, for example) may also affect the cellular response (52, 58). There is no doubt that the tumor microenvironment, including the composition of immune cells, can substantially affect the response and proliferation of tumor cells (59). Activation of TLRs in immune cells can result in a Th1 response, causing apoptosis (59). Further elucidation of TLRs and their downstream pathways is essential for understanding the complex interactions between cell proliferation and inflammation.

Lastly, we focused on the effects of P. aeruginosa on MAPK activation, showing that P. aeruginosa stimulation of KMM cells resulted in the activation of p38, ERK1/2, and JNK pathways. P. aeruginosa flagellin contributed to cell proliferation by activating the p38 and ERK1/2 pathways, and inhibition of the p38 and ERK1/2 pathway abrogated the enhanced cell proliferation in flagellin-stimulated KMM cells (Fig. 8C). The MAPK pathways play an important role in KSHV biology and likely in KS pathology. Primary infection of KSHV results in activation of p38, ERK1/2, and JNK activation, and inhibition of the MAPK pathways can reduce KSHV infectivity and induction of IL-6 (24, 60). The MAPK pathways also mediate KSHV reactivation (61). Our results identify the important role of P. aeruginosa-induced inflammation in cell proliferation and cellular transformation while providing further evidence on the therapeutic value of inhibiting the MAPK pathways in KS patients.

Overall, our study utilizes a clinically relevant, opportunistic bacterium to study the effect of inflammation on KSHV-transformed cells. We report that P. aeruginosa stimulation increases cell proliferation and cellular transformation in KSHV-transformed cells while having no significant effect on MM cells. These results emphasize that KSHV cellular transformation results in enhanced sensitivity to external stimuli, which may further increase cell proliferation and cellular transformation. As KS and HIV/AIDS patients are at an increased risk for opportunistic infection, it is critical to understand the effects of bacteria on KS pathogenesis. Our results indicate that elimination of certain bacterial infections identified to promote inflammation have a preventive value for KSHV-infected AIDS patients who are at a high risk for developing KS as well as a therapeutic value for AIDS-KS patients. Moreover, our study further dissects the specific P. aeruginosa PAMPs that contribute to cell proliferation, demonstrating that both LPS and flagellin can induce inflammation in KSHV-transformed cells. Besides LPS and flagellin, P. aeruginosa consists of many additional PAMPs that can induce inflammation, such as peptidoglycans and lipoproteins (62, 63). Analyzing the effects of these additional PAMPs on cell proliferation, by exploring the specific pathways and mechanisms of inflammatory induction, may clarify the correlative, additive, or synergistic effects as well as independent effects of these other PAMPS in inflammation processes in general as well as for inflammation and KS pathogenesis specifically.

## MATERIALS AND METHODS

### Cell culture.

Early passages (<20) of MM, KMM, BJAB, and KSHV-BJAB cells were grown as previously described (34, 64). All cell lines were routinely tested for mycoplasma contamination using a LookOut Mycoplasma quantitative PCR (qPCR) detection kit (MP0035-1KT; Sigma).

### Reagents.

Purified flagellin from P. aeruginosa (tlrl-pafla; InvivoGen) and ultrapure LPS from E. coli K-12 (tlrl-peklps; InvivoGen) were resuspended in water. CLI095 (tlrl-cli095; Thermo Fisher Scientific), SB203850 (NC9041893; Fisher), and U0126 (19-147; Sigma) were resuspended in dimethyl sulfoxide.

### Bacterial preparation.

Three P. aeruginosa strains were used for this study: P. aeruginosa (ATCC 15442), PAO1Δ*fliC* strain (JJH325), and PAO1. PAO1Δ*fliC* and PAO1 strains were kindly provided by Jennifer Bomberger (University of Pittsburgh), Joe Harrison (University of Calgary), and Matthew Parsek (University of Washington). P. aeruginosa strains were grown in LB broth (Sigma-Aldrich) overnight until an optical density (OD) value of 1.1. The culture was then washed three times in PBS by centrifuging at 3,200 × *g* for 10 min and then diluted with PBS to the specified concentrations (CFU per milliliter) for experiments. To confirm the accuracy of the bacterial concentration, the culture was serially diluted, grown on agar plates overnight, and analyzed for the number of colonies.

### Cell proliferation assay.

MM/KMM cells and BJAB/KSHV-BJAB cells were plated at a density of 20,000 and 10,000 cells/well (respectively) in 24-well plates for 16 h, treated with the indicated reagents, and counted using a Malassez chamber.

### Soft agar assay.

Colony formation in soft agar was carried out as previously described (34).

### RNA extraction and qRT-PCR.

Total RNAs were extracted with TRI Reagent (T9424; Sigma). Reverse transcription (RT) was performed with 1 μg of total RNA using SsoAdvanced universal SYBR green supermix (172-5272; Bio-Rad). cDNAs diluted 2 times were examined by qPCR with the KAPA SYBR fast qPCR kit (K4602; Kapa Biosystems) using specific primers for β-actin, IL-6, IL-1β, IL-18, TNF-α, and CXCL-1. The β-actin gene was used for loading calibration. All of the sequences of primers used for quantitative RT-PCR (qRT-PCR) are listed in Table 1.

**TABLE 1:** Sequences of primers used for qRT-PCR

Primer	Sequence (5′ to 3′)	
	Forward	Reverse
Rat β-actin	GGAAATCGTGCGTGACATTA	AGGAAGGAAGGCTGGAAGAG
Rat IL-6	TCCTACCCCAACTTCCAATGC	TTGGATGGTCTTGGTCCTTAG
Rat IL-1β	CATTGTGGCTGTGGAGAAG	ATCATCCCACGAGTCACAGA
Rat IL-18	GGCTCTTGTGTCAACTTCAAA	TTATCAGTCTGGTCTGGGATT
Rat TNF-α	TGAACTTCGGGGTGATCG	GGGCTTGTCACTCGAGTTTT
Rat CXCL-1	CATTAATATTTAACGATGTGGATGCGTTTCA	GCCTACCATCTTTAAACTGCACAAT

### Western blot analysis.

Western blot analysis was performed as previously described (65). Primary antibodies included mouse monoclonal antibodies to β-tubulin and rabbit polyclonal antibodies to glyceraldehyde-3-phosphate dehydrogenase (5174S; Cell Signaling Technology), JNK (9252S; Cell Signaling Technology), phospho-JNK (4668S; Cell Signaling Technology), p38 (8690S; Cell Signaling Technology), phospho-p38 (4511S; Cell Signaling Technology), ERK1/2 (4695S; Cell Signaling Technology), and phospho-ERK1/2 (4370S; Cell Signaling Technology).

### Statistical analysis.

Results are expressed as means ± standard errors from at least three independent experiments. Statistical analysis was performed using two-tailed *t* test, and a *P* value of ≤0.05 was considered significant.
